# Evolution of Female Preference for Younger Males

**DOI:** 10.1371/journal.pone.0000939

**Published:** 2007-09-26

**Authors:** Christopher W. Beck, Daniel E. L. Promislow

**Affiliations:** 1 Department of Biology, Emory University, Atlanta, Georgia, United States of America; 2 Department of Genetics, University of Georgia, Athens, Georgia, United States of America; University of Uppsala, Sweden

## Abstract

Previous theoretical work has suggested that females should prefer to mate with older males, as older males should have higher fitness than the average fitness of the cohort into which they were born. However, studies in humans and model organisms have shown that as males age, they accumulate deleterious mutations in their germ-line at an ever-increasing rate, thereby reducing the quality of genes passed on to the next generation. Thus, older males may produce relatively poor-quality offspring. To better understand how male age influences female mate preference and offspring quality, we used a genetic algorithm model to study the effect of age-related increases in male genetic load on female mate preference. When we incorporate age-related increases in mutation load in males into our model, we find that females evolve a preference for younger males. Females in this model could determine a male's age, but not his inherited genotype nor his mutation load. Nevertheless, females evolved age-preferences that led them to mate with males that had low mutation loads, but showed no preference for males with respect to their somatic quality. These results suggest that germ-line quality, rather than somatic quality, should be the focus of female preference in good genes models.

## Introduction

Various models of sexual selection have been put forward to explain the origin and maintenance of elaborate male secondary sexual traits. These models focus on one or more of five possible factors that shape female preference, including direct benefits to the female, Fisher's runaway process, sensory bias, sexual conflict, and indirect benefits to offspring (so-called ‘good genes’ models) [1], [2]. According to this last model, females evolve choosiness as a way of maximizing the quality of the genes that are passed on from mates to offspring. Under a good genes model, we expect a positive correlation between male quality and fitness components in the offspring.

Models for good genes processes assume that females can use some phenotypic trait, such as brightly colored tail feathers or an elaborate courtship dance, as an indicator of underlying male quality [e.g.], [ 3], [4]–[6]. In recent years, biologists have considered the possibility that age itself may be an accurate indicator of male quality [7]–[11]. If males exhibit heritable variation for survival rate, then older males will be expected to have higher than average quality (or lower than average intrinsic mortality). Thus, male age has been considered to be a reliable indicator of mutation load [1], [12]–[14].

Formal models of age-specific mate preference have found that when females choose mates based on age cues alone, females will generally evolve a preference for older males [7], [8], [10], [but see 15]. However, the benefits of mating with older males could potentially be mitigated by the deleterious effects of *de novo* germ-line mutations [16].

Several studies have recognized a role for mutation in sexual selection. In good genes models, variation in mutation load among males in a population is essential for female mate choice to evolve [4], [17]–[20]. Similarly, sperm competition may lead to the evolution of relatively high male mutation rates, and so drive an overall increase in mutation load in both sexes [16]. At the same time, sexual selection may reduce mutation load if females choose to mate with males with relatively low mutation loads [21]–[23] or if male fitness is more greatly affected than female fitness by deleterious mutations [24].

These mutations also might be important in shaping female preference for old males. While the ‘somatic’ genetic quality of an individual may not change with age, the gamete mutation load of an individual can increase with age if germ-line mutation probability increases with age. Because germ-line mutations occur during division of germ cells, the probability of mutations increases with the number of divisions. Theory predicts an increase in germ-line mutations with age [25], and data on paternally-inherited genetic disorders in humans support this prediction [26]–[30]. These data suggest that male quality and gamete mutation load must be distinguished in models of sexual selection [31].

Thus, two potentially opposing forces act on aging males. On the one hand, within-cohort selection should lead to higher relative fitness in older males. On the other, if germ-line mutations increase with age, older males could produce relatively unfit offspring. Taken together, we might expect to see a negative correlation between male quality and gamete load among individuals within a population. In light of this, we need to determine if female preference on male age is shaped primarily by the effects of within-cohort selection (in which case females should prefer to mate with older males) or by *de novo* mutations in gametes (in which case females should prefer younger males).

All this points to a variety of conflicting forces. Increased germ-line mutations can enhance the opportunity for sexual selection, while sexual selection can reduce the mutation load. Higher mortality rates can increase preference for older males by increasing within-cohort selection, but preference for older males can lead to reduced mortality rates [8]. And of course, both higher mutation rates and higher extrinsic mortality can lead to higher rates of aging [32], [33]. Given these conflicting forces, the model described here attempts to better understand the evolution of female preference for male age. We use a simulation model based on a genetic algorithm [8] to determine (a) whether increased mutation probability with male age will influence male gamete mutation load, and consequently, female preference based on male age; and (b) whether female preference based on male age can lead to a reduction in average mutation load for males.

## Methods

### Genetic algorithm

To examine the interaction between male gamete mutation load and female preference based on male age, we modified our previous genetic algorithm [8] to include age-specific mutation probability in males. Rather than defining a particular preference function based on male age, the algorithm allows female preference for males in each age class to evolve freely, thus allowing us to determine the optimal preference function. The basic structure of the algorithm is described below.

Each haploid individual in the population is defined by ten preference loci and ten mortality loci. Each preference locus (*a_i_*) can take on any value on the continuous scale from 0 to 1. This value is the probability that a female who encounters a male of age *i* will mate with that male. Ten mortality loci (*m_k_*) have either of two alleles at each locus, −1 or 1, which decrease and increase mortality, respectively. Individual mortality rates are determined by a combination of a genotype-independent mortality function and the ten mortality loci that can differ among individuals, based on the following equation:
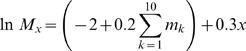
(1)where *M_x_* is the intrinsic rate of mortality at age *x*. This model of mortality is supported by the work of Promislow & Tatar [34] who note that mutational and environmental alterations appear to be more likely to affect the age-independent rather than the age-dependent component of mortality. The probability of an individual surviving is given by 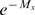. This age-specific survival curve is an intrinsic property of each individual, based on the specific set of haploid m-loci inherited from that individual's mother and father. This survival function is what we refer to as the individual's phenotype. Thus, a high-quality phenotype is one with a relatively high survival rate (i.e., low *M_x_*).

Each cycle of the model includes four components—i) mate choice; ii) reproduction and mutation with Mendelian inheritance; iii) adult age-specific, density-independent mortality; and iv) juvenile, density-dependent mortality. For mate choice, each female randomly selects a male from the population and decides whether to mate with him based on his age *i* and the value at her preference locus, *a_i_*, for males of that age. If a female chooses not to mate with the selected male, she is allowed to select additional males until she finds one she prefers or until she has encountered ten males. If the tenth male is not chosen by the female, she is mated to this male. Females only mate once per cycle, but males can mate multiple times. In simulations with random mating, mate choice is skipped and females are mated to randomly selected males, irrespective of the values at the females' preference loci.

After mating, four offspring of each sex are produced by each mating every cycle. Offspring inherit each mortality locus from either the mother or father with equal probability. Mortality loci inherited from mothers mutate with a probability of 0.01. Those inherited from fathers mutate with a probability μ*_x_* (see equations 2 and 3, below), which depends on the father's age and the mutation probability function. Mutations were biased such that only increases in mortality were possible. The mortality of individual adults is determined by their age and alleles at the mortality loci. All 10-year-old individuals are removed in the following cycle. Juvenile mortality is density-dependent. Mortality of offspring is determined based on mortality alleles and equation 1. Then, sufficient offspring are selected at random from the surviving offspring, such that the total adult population size remains constant. We use a large population of 10,000 adults to minimize the effects of genetic drift on the evolution of the female preference function. A 50∶50 sex ratio is maintained in the adult population.

Whether and how mutation probability changes with male age depends on the type of mutation [30]. For example, in human populations, mutations associated with achondroplasia increase in frequency with male age, whereas aneuploidies do not [30]. For mutations that increase in probability with male age, the increase can either be linear [27] or non-linear [28], [30]. A linear increase in mutation probability with male age could be attributed to continual stem cell divisions in male germ cells [35]. In contrast, non-linear increases in mutation probability with male age might be due to selection for mutations in the germline prior to meiosis [35], [36]. When mutation probability increases in a non-linear fashion with respect to male age, Crow [26] found that an exponential function with a cubic term best describes the increase in mutation rate with male age in humans. To capture patterns derived from these two observations, we analyzed two modes of age-related change in mutation frequency. First, we examined simulations in which μ*_x_*, the mutation probability at age *x*, increases as a linear function of male age,
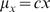
(2)


where *c* is the germ-line mutation rate at age 1. As an alternative, we examined simulations in which μ*_x_* increases as a cubic function of male age
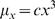
(3)as this is the simplest exponential function with a cubic term.

For both mutation probability functions, we examined three different coefficients such that the maximum mutation probabilities were 0.01, 0.05, and 0.1, respectively. We refer to this maximum mutation proability as “mutation rate” throughout. A set of control runs assumed that mutation rate was a constant value of 0.01, which previously was found to maintain variation among males at mutation-selection balance [8]. For the smallest coefficient, the mutation probability of the oldest males (10-year-old) is equal to that of females (0.01). For the largest coefficient, the mutation probability of 1-year-old males is equal to that of females.

Whereas a male's somatic quality is determined by the haploid genotype he inherits from his parents, his gametic quality depends on both his inherited genotype, and the degree to which newly-acquired germ-line mutations will reduce the quality of that genotype.

### Analysis of Simulations

For each set of parameters, the simulation was run for 320,000 cycles, and each simulation was repeated 8 times. From these replicate simulations, we were able to determine the mean and standard error of the female preference, male somatic quality, relative male somatic quality, male gamete mutation load, and relative male gamete mutation load for each male age class. Male somatic quality was calculated as the sum of the beneficial mutations at the ten mortality loci. In contrast, *L_x_*, the expected gamete mutation load for a male of age *x* with *n* deleterious mutations and (10 − *n*) advantageous alleles at the ten mortality loci, is given by
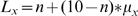
(4)with a mutation shifting a mortality locus from *m* = −1 to +1. Absolute mutation load can range from zero (no load) to ten in our simulations. To determine the relative somatic quality and relative gamete mutation load of males of a particular age for a given replicate, we calculated average values of somatic quality and gamete mutation load, respectively, for males within a replicate and divided by the highest average male quality or load within a replicate. Thus, the data from each replicate simulation was represented by a single data point for each age class.

For each mutation rate, we determined the relationship between female preference and male gamete mutation load using the partial correlation between average female preference and average relative gamete mutation load for males across age classes, controlling for the effect of relative male somatic quality. Similar analyses were carried out to examine the relationship between female preference and male somatic quality after controlling for the effect of relative gamete mutation load.

## Results

**Figure 1:**
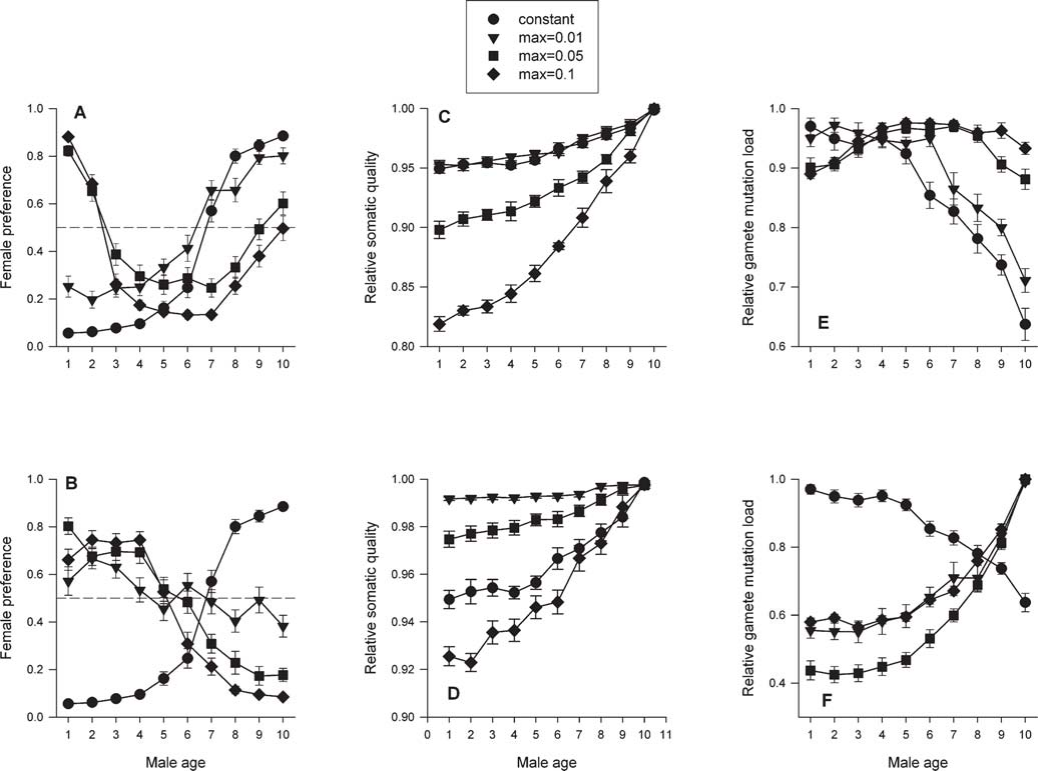
The effect of increasing mutation probability with male age on female preference based on male age (A, B), relative male somatic quality (C, D), and relative male gamete mutation load (E, F). Mutation probability increased either as a linear (A, C, E) or as a cubic (B, D, F) function of male age. Values represent the mean±1 standard error among eight replicate simulations after 320,000 cycles. Dashed line is the value for female preference expected by chance alone.

As reported previously [8], when mutation probability was constant across all male age classes, females exhibited a preference for older males and a bias against younger males (Fig. 1A). In contrast, when mutation probability increased with male age, females generally preferred younger males (Fig. 1A, B). However, mutation probability function influenced female preference based on male age (Fig. 1A, B). When mutation probability increased linearly with male age, females generally evolved a preference for younger males and avoided intermediate age males (Fig. 1A). In contrast, when mutation probability increased as a cubic function of male age, females exhibited a preference for younger males and against older males (Fig. 1B). For both mutation probability functions, viability selection led to similar changes in relative somatic quality with respect to male age (Fig. 1C, D). The youngest males were of the lowest somatic quality, whereas the oldest males were of the highest quality. However, the relationship between relative gamete mutation load and male age differed considerably between the two mutation probability functions (Fig. 1E, F). Linear increases in mutation probability with male age did not outweigh the effect of viability selection on male quality. As a result, the gamete mutation load of the oldest males was lower than that of intermediate-aged males. Yet, when mutation probability increased as a cubic function of male age, gamete mutation load was highest in the oldest age classes, despite the fact that these males were of the highest somatic quality.

**Figure 2:**
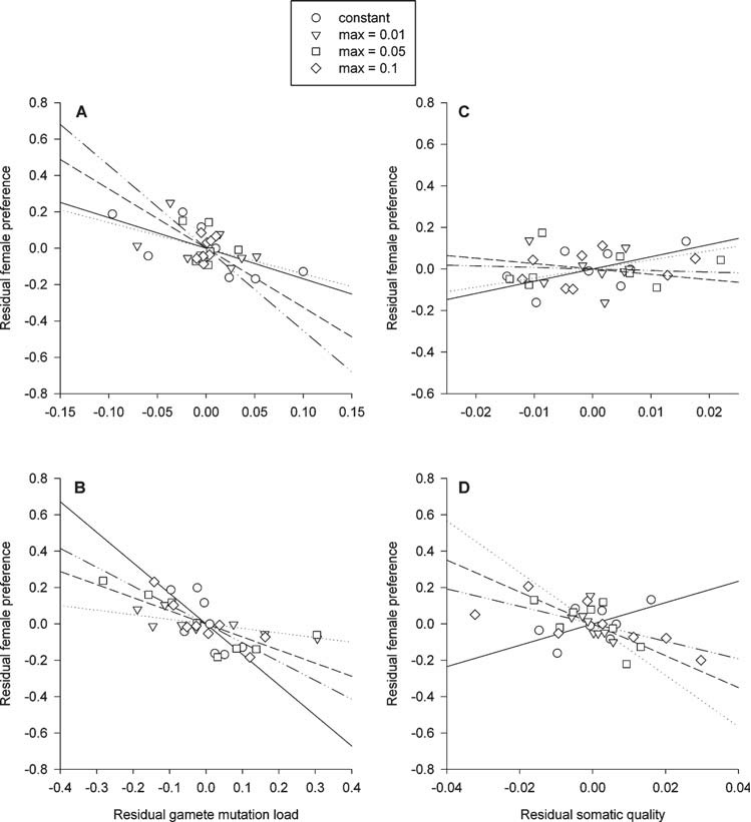
The relationship between female preference and relative average male gamete mutation load, controlling for relative average male somatic quality (A, B) or male quality, controlling for relative average male gamete mutation load (C, D) for different mutation rates. Mutation probability increased either as a linear (A, C) or as a cubic (B, D) function of male age. Relationships based on eight replicate simulations of 320,000 cycles across all ten age classes (N = 80). Based on partial correlation analyses, all relationships are significant except when the maximum mutation rate was 0.01. For clarity, individual data points were ranked by the independent variable and placed in bins of ten. The data points on the graphs represent the averages of the individual data points with each bin. The regression lines are based on all 80 data points for a given mutation rate. Constant (solid line), max = 0.01 (dotted line), max = 0.05 (dashed line), max = 0.1 (dashed and dotted line).

By comparing female preference functions based on male age (Fig. 1A, B) with how relative male gamete mutation load changes with male age (Fig. 1C, D), it becomes clear that females generally prefer males with the lowest relative gamete mutation load and avoid males with the highest relative gamete mutation load. As a result, in most cases, female preference was negatively correlated with relative gamete mutation load after controlling for relative male somatic quality, independent of mutation probability function (Fig. 2A, B). Interestingly, opposite to what would be predicted, when mutation probability increased with male age, female preference was negatively correlated with relative male somatic quality, after controlling for relative gamete mutation load (Fig. 2C, D). Only when mutation probability was constant with male age were female preference and relative male somatic quality positively correlated.

Female preference functions also were influenced by mutation rate. For both mutation probability functions, female preference functions were different when mutation rate was low, as compared to intermediate and high mutation rates. When mutation probability was a linear function of male age, female preference functions did not differ between simulations with a constant mutation rate and those with a low mutation rate (Fig. 1A), because gamete mutation load changed with male age similarly in both cases (Fig. 1C). In contrast, when mutation probability changed as a cubic function of male age and mutation rate was low, females did not distinguish among males of different ages. The absence of female preference based on male age in this case might have been due to the low average absolute mutation load across all age classes (0.128). For intermediate and high mutation rates, the average absolute mutation loads across all age classes were 0.438 and 1.07, respectively.

We also examined how the effect size of a mutation influences female preference (see Text S1). Although changes in the effect size of mortality loci resulted in slight changes in the shape of the female preference functions (Fig. S1,S2), the relationships between female preference and either relative gamete mutation load or relative somatic quality of males were fundamentally the same (Table S1).

**Figure 3:**
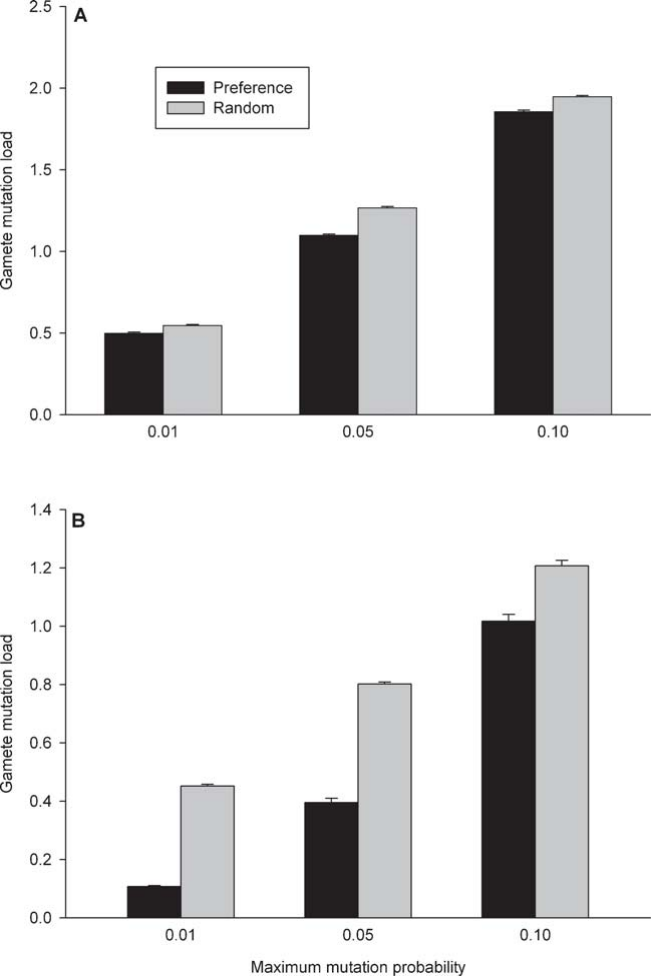
Effect of female preference on male gamete mutation load across all age classes for linear (A) and cubic (B) mutation probability functions. Mutation load represents the mean±1 standard error among eight replicate simulations after 320,000 cycles. Mutation load is significantly lower when females exhibit a preference in all cases.

Our model allowed for a dynamic interaction between mutation load and female preference. In particular, we found that female preference based on male age led to a reduction in male gamete mutation load as compared to random mating for both mutation probability functions (Fig. 3). The average reduction was greater for cubic increases in mutation probability (4.8 standard deviations) than for linear increases in mutation probability (1.6 standard deviations). In addition, the effects of female preference on male gamete mutation load depended on mutation rate. Female preference resulted in the greatest decrease in male gamete mutation load at intermediate (2.5 standard deviations) and low (7.0 standard deviations) mutation rates for linear and cubic mutation probability functions, respectively (Fig. 3).

## Discussion

Previous models for the evolution of female preference based on male age have showed that in most cases, females will evolve a preference for intermediate-aged and older males and a bias against younger males when mutation probability is constant with age [7], [8], [10]. In human populations, whether due to female preference or simply to cultural changes in our own lifetime, over the last several decades we have seen the number of men between 35 and 49 years-old fathering children increase markedly [37]. Previous evolutionary theory would predict that these men would be of higher genetic quality than younger men [1], [12]–[14], [22]. Yet, as our model suggests, this bias towards older men both in models of sexual selection, and in the real world of human reproduction, may bring with it some unintended costs.

Recent studies of a variety of paternally-inherited genetic disorders suggests that the incidence of these disorders increases with paternal age [28]–[30], [38]. In fact, even in healthy individuals, daughters of older fathers have been shown to have shorter lifespans than daughters of younger fathers [39]. Thus, older males, or at least their sperm, might be of lower genetic quality than that of younger males. In our model, we made the assumption that mutation probability, and thus gamete mutation load, will increase with male age. Based on this same assumption, Ellegren recently predicted that females should evolve a preference for younger males [16]. Indeed, we found that females generally exhibited a preference for younger males and a bias against either intermediate-age or older males. Due to this preference, offspring of females that preferred younger males were less likely to inherit deleterious mutations. In other words, females can show a strong preference based on male age-specific gamete mutation load even if they have no way of actually measuring gamete mutation load. If females can differentiate among males based on age, they will evolve a preference that is consistent with their choosing males with the lowest gamete mutation load.

Most good genes models of sexual selection assume that the somatic quality of a male and the quality of his gametes are positively correlated. As a result, female preference should be positively correlated with male somatic quality, if female preference evolves as a result of good genes. In contrast with this prediction, we found that female preference was often negatively correlated with male somatic quality when mutation probability increased with male age. The contrasting results with respect to the quality of males and the genetic quality of their gametes reinforces the idea that male gamete quality may be a stronger driving force than male somatic quality in the evolution of female choice [31]. Furthermore, our results are the first of which we are aware that support the suggestion that in studies of sexual selection, genetic quality of males is best estimated as the breeding value for total fitness, rather than estimates of specific phenotypic fitness components [40].

The results of our model are consistent with those of recent empirical studies. First, females of several species have been shown to be able to distinguish among males of different ages [41], [42], which is a pre-requisite for the evolution of female preference based on male age. In addition, empirical studies of several species have found that females do not prefer to mate with older males [see Table 1 in 43], and may in fact pay a fitness cost when mating with those males. For example, in the sandfly *Lutzomyia longipalpis*, eggs sired by older males have decreased hatching rates, and females avoid those males [44]. In other species in which offspring of older males have lower fitness [e.g. Drosophila 11], [45], we might expect females to evolve preferences for younger males, or at a minimum, to avoid mating with older males.

Although our results suggest that females should avoid mating with older males, in some species females seem to prefer older males, while in others, females do not distinguish among males based on age [reviewed in 43]. Females may prefer older males for several reasons. First, older males may provide greater direct benefits than younger males (e.g., territories, parental care). These direct benefits might outweigh the indirect costs of poorer gamete quality. Empirical studies suggest that direct benefits of female mate choice are more important than indirect benefits in some taxa [46]–[48]. However, our model assumed no direct benefits. Second, if the probability of germline mutations does not increase with male age, then the gametes of older males may be of higher quality, which would lead to preference for older males [8]. Third, in some species, such as *Drosophila melanogaster*, females pay a mating cost in terms of lowered fecundity [49]. If the manipulative ability of accessory gland proteins in older males is less than that in younger males, mating with older males might lead to lower costs to females.

The absence of female preference based on male age also may be due to several factors. First, females may not be able to distinguish the age of potential mates. Although possible, this explanation seems unlikely, as previous studies from a diverse array of species have found that females can distinguish among males based on age [41], [42]. Furthermore, male signals are predicted [3], [50] and have been found to be reliable indicators of male age [51]–[62]. Second, if the costs associated with exhibiting a preference are high, preference may not evolve [7], [8], [10], [63]. Finally, our model suggests that if absolute mutation load is low across all age classes, females will not evolve a preference based on male age. In this case, gametes of males of all age classes are of high quality, so there is little selection on females to prefer males of a particular age.

Hansen and Price [25] suggested that the evolution of female preference based on male age might depend on the effect of a given deleterious mutation on offspring fitness. However, we found that the relationship between female preference and relative gamete mutation load was independent of the size of the effect of a mutation. Therefore, the evolution of female preference due to “good genes” is independent of the effect of mutation of those genes. Because our populations are in mutation-selection balance and female preference is negatively correlated with mutation load, this result is in line with classical theory that suggests that mutation load is independent of effect size [64], [65].

Kirkpatrick and Hall [66] suggest that evolution of female preference under a good genes model is more likely with X-linked preferences or autosomal displays. However, Mank et al. [67] found no correlation between sex chromosome systems and sexually-selected traits in fishes. Our model is haploid; thus, we were unable to explore the effect of male and female heterogamety on the evolution of female preference based on male age. This would be an interesting direction for future research.

Although the main intent of our simulations was to examine the effect of age-related increases in germ-line mutation rate on the evolution of female preference, our results also point to a role for female preference on gamete mutation load. We found a significant decrease in mutation load in populations in which females exhibited a preference based on male age. This observation was in line with previous models that have predicted that female mate choice should reduce mutation load [21]–[23]. This reduction in mutation load could lead to lower mortality rates at all ages. These results therefore suggest how female preference might play an important role in the evolution of aging [68].

In general, our results suggest that if the rate of germ-line mutations increases with age, then females should evolve a preference for younger males. By mating with younger males, females are able to avoid mating with males with low quality sperm. In addition to being of lower genetic quality, older males might produce less sperm, thereby decreasing fertilization success and female fecundity [e.g.], [69,70]. Either case might lead to sexual conflict over mating [71]. Importantly, our results show that male somatic quality and male sperm quality are not always correlated, and that females should evolve to choose males based on sperm quality rather than somatic quality. Although we found a general pattern of female preference for younger males independent of mutation probability function, our results do suggest the importance of accurate estimates of the age-related change in mutation rate in males if we are to predict how female preference based on male age will evolve.

## Supporting Information

Text S1 Supporting text. Methods associated with data presented in Fig. S1,S2 and Table S1.
(0.01 MB PDF)

Figure S1 The effect of mutation probability increasing as a cubic function of male age on female preference based on male age (A, B) and relative male gamete mutation load (C, D) when mutations have small effects on mortality. Data are from simulations either with 20 mortality loci each with half of the effect of mortality loci in the standard simulations (A, C) or from simulations with 40 mortality loci each with one-quarter of the effect of mortality loci in the standard simulations (B, D). Values represent the mean±1 standard error among 8 replicate simulations after 320,000 cycles. Dashed line is the value for female preference expected by chance alone.
(0.02 MB PDF)

Figure S2 The effect of mutation probability increasing as a cubic function of male age on female preference based on male age (A, B) and relative male gamete mutation load (C, D) when mutations have large effects on mortality. Data are from simulations either with 5 mortality loci each with twice the effect of mortality loci in the standard simulations (A, C) or from simulations with 10 mortality loci each with twice the effect of mortality loci in the standard simulations (B, D). Values represent the mean±1 standard error among 8 replicate simulations after 320,000 cycles. Dashed line is the value for female preference expected by chance alone.
(0.02 MB PDF)

Table S1 Partial correlations between female preference and relative gamete mutation load or relative somatic quality for different maximum mutation probabilities and mutation effect sizes
(0.01 MB PDF)
