# Supplementary material for: Evolution of Female Preference for Younger Males
Source: PLoS One. 2007 Sep 26;2(9):e939. doi: 10.1371/journal.pone.0000939 (PMC1976549; doi:10.1371/journal.pone.0000939)
Supplement: Text S1 — Supporting text. Methods associated with data presented in Fig. S1,S2 and Table S1. (0.01 MB PDF) [file pone.0000939.s001.pdf]

Text S1

Supporting text

To decrease the effect of deleterious mutations in comparison to baseline simulations, we increased the number of mortality loci. For simulations in which we used 20 mortality loci instead of 10 mortality loci, the effect of each locus was one-half that of the effect of each locus in the baseline simulations. Similarly, for simulations in which we used 40 mortality loci, the effect of each locus was one-quarter that of the effect of each locus in the baseline simulations. To increase the effect of deleterious mutations, we used two approaches. First, we decreased the number of mortality loci to five, such that the effect of each locus was twice that of the effect of each locus in the baseline simulation. Second, we kept the same number of loci, but doubled the effect of each locus on mortality rate. In all of these simulations, both the mean and variance in mutation load changes in comparison to the baseline simulations. Therefore, any differences as compared to the baseline simulations could be due to either factor.
