# Supplementary material for: Evolution of Female Preference for Younger Males
Source: PLoS One. 2007 Sep 26;2(9):e939. doi: 10.1371/journal.pone.0000939 (PMC1976549; doi:10.1371/journal.pone.0000939)
Supplement: Figure S2 — The effect of mutation probability increasing as a cubic function of male age on female preference based on male age (A, B) and relative male gamete mutation load (C, D) when mutations have large effects on mortality. Data are from simulations either with 5 mortality loci each with twice the effect of mortality loci in the standard simulations (A, C) or from simulations with 10 mortality loci each with twice the effect of mortality loci in the standard simulations (B, D). Values represent the mean±1 standard error among 8 replicate simulations after 320,000 cycles. Dashed line is the value for female preference expected by chance alone. (0.02 MB PDF) [file pone.0000939.s003.pdf]

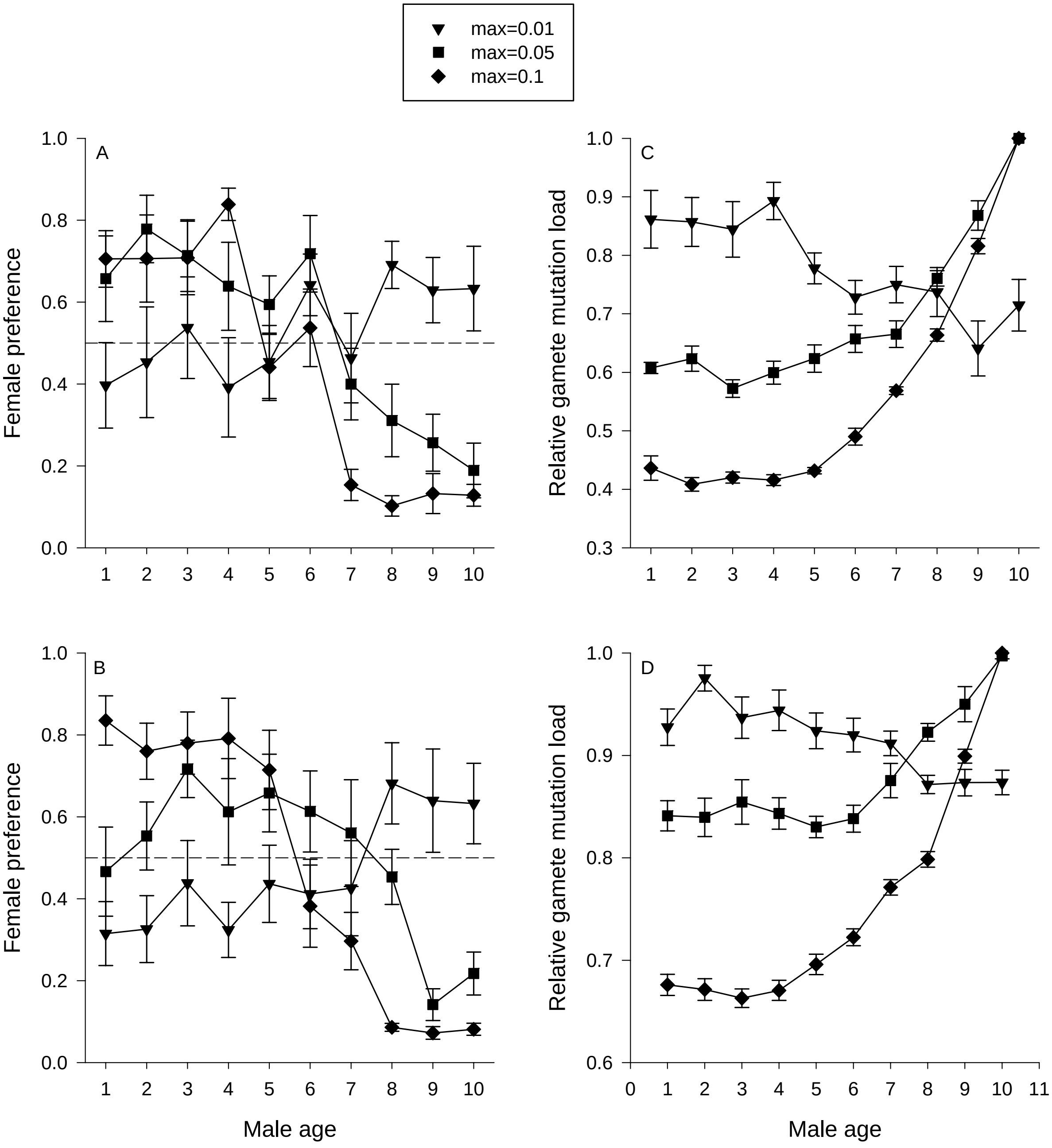

Figure S2: The effect of mutation probability increasing as a cubic function of male age on female preference based on male age (A, B) and relative male gamete mutation load (C, D) when mutations have large effects on mortality. Data are from simulations either with 5 mortality loci each with twice the effect of mortality loci in the standard simulations (A, C) or from simulations with 10 mortality loci each with twice the effect of mortality loci in the standard simulations (B, D). Values represent the mean ± 1 standard error among 8 replicate simulations after 320,000 cycles. Dashed line is the value for female preference expected by chance alone.
