# Supplementary material for: Evolution of Female Preference for Younger Males
Source: PLoS One. 2007 Sep 26;2(9):e939. doi: 10.1371/journal.pone.0000939 (PMC1976549; doi:10.1371/journal.pone.0000939)
Supplement: Table S1 — Partial correlations between female preference and relative gamete mutation load or relative somatic quality for different maximum mutation probabilities and mutation effect sizes (0.01 MB PDF) [file pone.0000939.s004.pdf]

Table S1. Partial correlations between female preference and relative gamete mutation load or relative somatic quality for different maximum mutation probabilities and mutation effect sizes

		Relative gamete mutation load			Relative somatic quality		
Effect size	Number of loci	max = 0.01	max = 0.05	max = 0.1	max = 0.01	max = 0.05	max = 0.1
Large	5	0.06	-0.15	-0.30**	0.23*	-0.46***	-0.31**
Large	10	0.10	-0.25*	-0.38**	0.26*	-0.36**	-0.37**
Small	20	-0.27*	-0.27*	-0.32**	0.29*	-0.32**	-0.33**
Small	40	-0.08	-0.20	-0.13	0.25*	-0.20	-0.41***

*P<0.05, **P<0.01, **P<0.001
